# A Review of African Americans’ Beliefs and Attitudes About Genomic Studies: Opportunities for Message Design

**DOI:** 10.3389/fgene.2019.00548

**Published:** 2019-06-14

**Authors:** Courtney L. Scherr, Sanjana Ramesh, Charlotte Marshall-Fricker, Minoli A. Perera

**Affiliations:** ^1^Department of Communication Studies, Center for Communication and Health, Northwestern University, Chicago, IL, United States; ^2^Department of Pharmacology, Center for Pharmacogenomics, Feinberg School of Medicine, Chicago, IL, United States

**Keywords:** African American, genomics, health communication, pharmacogenomics, precision medicine, recruitment

## Abstract

Precision Medicine, the practice of targeting prevention and therapies according to an individual’s lifestyle, environment or genetics, holds promise to improve population health outcomes. Within precision medicine, pharmacogenomics (PGX) uses an individual’s genome to determine drug response and dosing to tailor therapy. Most PGX studies have been conducted in European populations, but African Americans have greater genetic variation when compared with most populations. Failure to include African Americans in PGX studies may lead to increased health disparities. PGX studies focused on patients of African American descent are needed to identify relevant population specific genetic predictors of drug responses. Recruitment is one barrier to African American participation in PGX. Addressing recruitment challenges is a significant, yet potentially low-cost solution to improve patient accrual and retention. Limited literature exists about African American participation in PGX research, but studies have explored barriers and facilitators among African American participation in genomic studies more broadly. This paper synthesizes the existing literature and extrapolates these findings to PGX studies, with a particular focus on opportunities for message design. Findings from this review can provide guidance for future PGX study recruitment.

## Introduction

Precision Medicine (PM) refers to the targeting of therapies according to an individual’s, genetics, lifestyle or environment and holds immense promise to improve population health outcomes (Khoury et al., 2016). A branch of precision medicine, pharmacogenomics (PGX) is the study of genetic information to determine individual response (e.g., efficacy/toxicity) to pharmaceutical agents with the goal of developing safe and effective medications and dosage that can be tailored based on an individual’s genetics (Lee, 2003; Empey, 2016). In order to draw conclusions about gene interactions and genetic variation within and across ancestries, substantial and diverse patient data are needed (Jaffe, 2015; Khoury et al., 2016). To date, most PGX participants are of European ancestry (Perera et al., 2014). However, African Americans have greater genetic variation than European populations, therefore, results from existing PGX studies may not be as predictive in African American populations (Johnson et al., 2011; Perera et al., 2014). Under-representation of African American populations impairs the ability to translate PGX findings into clinical care, and will ultimately result in increased health disparities (Perera et al., 2014).

The challenge of recruiting minority populations likely stems from historic and contemporary mistreatment. For example, the Tuskegee Syphilis study has had a lingering effect on African Americans trust of medical institutions and research (Gamble, 1997). In addition to historic mistrust related to clinical research more broadly, genomic studies are further problematized due to concerns about personal identification, disenfranchisement stemming from genomic-based policies, and the potential threat of eugenics (Jackson, 1999). Furthermore, concerns about the inability for genomic research to address issues of social justice, and potentially exacerbate issues of health disparities remain (Jackson, 1999). Although few studies have examined the recruitment of African Americans to PGX studies, several have reported African American recruitment for genetic studies or biobanks (which we hereinafter refer to as genomic studies for simplicity).

Prior studies have reported demographic differences, for example, that African Americans are less likely to participate in research that includes a DNA sample or a biopsy compared with whites (Dye et al., 2016; Moledina et al., 2018). However, other studies have reported conflicting findings related to demographic factors influencing participation. One study related to prostate cancer genomics compared African American participants with white participants and found African American participants were younger, less educated, lower income, and less likely to be married compared with white participants (Patel et al., 2012). However, a different study found that African American women who provided a saliva sample for genomic research were older, regularly took a multivitamin, had a physician visit in the previous year, and reported a history of breast colorectal, or cervical screening compared with African American women who did not provide a saliva sample (Adams-Campbell et al., 2016). While demographic differences are useful in the categorization of participants, they do not provide useful insight for recruitment efforts.

Literature on recruitment efforts often describe community-based approaches (CBA) to engage participants in genomic studies by emphasizing intentional and meaningful community member engagement throughout the research process (Israel et al., 1998; Vadaparampil and Pal, 2010; Kiviniemi et al., 2013; Ochs-Balcom et al., 2015; McNeill et al., 2018). However, CBA focus on broad methods for recruitment and less on message content. Existing studies also have reported on the use of educational materials and seminars to improve African American recruitment (Skinner et al., 2008; Halverson and Ross, 2012; Rodriguez et al., 2016; Radecki Breitkopf et al., 2018). Studies found pre-post increases in knowledge about genomic studies, more favorable attitudes (Patel et al., 2018) and less negative affect (Kiviniemi et al., 2013) after receiving an educational intervention. However, random control trials and other studies employing pre-post assessment found no changes in attitudes about genomic research because of educational interventions (Skinner et al., 2008; Halverson and Ross, 2012). Such findings are not surprising because attitudes do not correlate with knowledge, but are shaped by values and beliefs (Grimshaw et al., 2002; Marteau et al., 2002; Fishbein and Yzer, 2003). Therefore, recruitment messages which address beliefs and attitudes related to participation in PGX studies, in addition to providing education, may speak more directly to African Americans’ concerns, and may more consistently improve recruitment efforts (Scherr et al., 2017).

Existing literature regarding African Americans’ beliefs and attitudes about genomic studies is disparate, and sometimes conflicting. Aggregating existing information provides an opportunity to reflect on current findings and potentially guide recruitment message strategies. Therefore, the objective of this paper is to systematically review qualitative and quantitative literature on African Americans’ beliefs and attitudes about genomic studies that may influence their decision to participate. We synthesized results from this review to highlight opportunities for the design of genomic study recruitment messages.

## Materials and Methods

### Study Design

Studies that provided insight regarding African Americans’ beliefs and attitudes toward participation in biobanks or genomic studies (inclusive of genetic or PGX) were included in this review. We focused on biobank and genomic studies because, to the best of our knowledge, no studies have exclusively explored African Americans’ beliefs and attitudes about PGX. Qualitative and quantitative studies with original empirical data were included, but conference abstracts, reviews, commentaries, editorials, legal opinions, letters to the editors, case studies, dissertations, and thesis studies were excluded. Given the potential influence of historical context, we excluded studies conducted outside the United States. We were interested in genetic studies that may be able to provide information on the treatment of chronic adult onset conditions; therefore, we excluded studies related to behavioral, developmental, or mental health genomics because we believed contextual factors (e.g., stigma, environment) could impact the results of such studies. We also excluded studies that explored medical professionals’ attitudes or beliefs about genomic studies because, while valuable, their attitudes and beliefs may be influenced by their additional education and training. We excluded studies that included less than 13% African Americans as a proportion of the total sample, which is consistent with the proportion of African Americans in the United States population. Finally, we excluded studies in which we could not distinguish African Americans’ responses from the responses of other study participants. Genomic studies have been conducted over a relatively limited period; therefore, we included all studies accepted for publication up to July 25, 2018 in this review.

### Information Sources and Search

A study team member worked with a University librarian and searched PubMed, Scopus, Web of Science, Embase, and Google Scholar for relevant citations. The search string was as follows: “African American” OR Black AND “genetic research” OR “pharmacogenomics research” OR “genomic research” OR “personalized medicine” OR “precision medicine” AND “study recruitment” OR “research participation.” The initial search returned 1,179 total citations: 15 from PubMed, 14 from Scopus, 133 from Web of Science, 26 from Embase, and 990 from Google Scholar. After consolidating the lists, we removed 109 duplicate citations, for a final sample of 1,070 citations.

### Study Selection

We screened studies for eligibility by conducting a review of the study titles, followed by an abstract review, and finally a full text review. Reviewers were instructed to be conservative in their exclusion; when uncertain, the study was retained. One study team member conducted the review of titles and excluded those that did not meet eligibility criteria. A second team member reviewed 20% of the titles to confirm exclusion criteria reliability. Kripendorf’s α = 0.73 was achieved, an acceptable level of reliability (Krippendorff, 2004). Next, two study team members split the remaining abstracts evenly for review, and excluded those which did not meet eligibility criteria. Twenty percent of the abstracts overlapped for reliability calculation, and α = 0.86 was achieved. Finally, one study team member reviewed 92% and another study team member reviewed 28% of full text and excluded those that did not meet eligibility criteria. Twenty percent of the full text overlapped to calculate reliability, and α = 0.85 was achieved.

### Data Analysis

One study team member reviewed the final studies included in the analysis to extrapolate information including the study design, the population setting, the total sample size, the sample race, and age. Two study team members conducted thematic analysis of the articles using MAXQDA to manage the data (VERBI Software, 2018).

## Results

**FIGURE 1:**
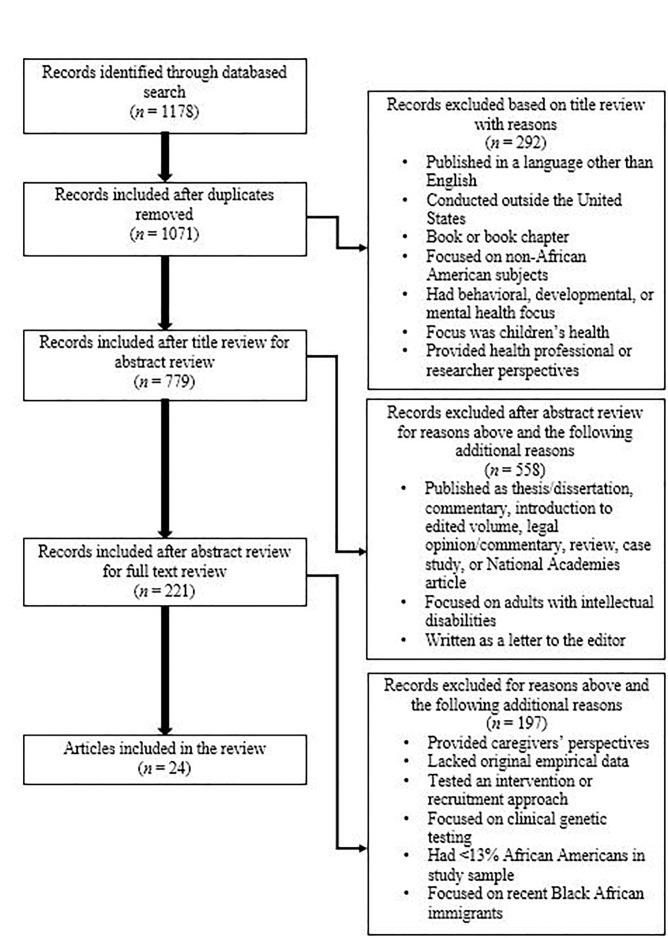
Exclusion process.

Of the 1,070 total titles screened, we removed 292 based on the title review, 558 based on the abstract review, and 197 based on the full text review, for a final sample of 24 articles (see Figure 1).

### Review of Studies

Our review of the literature (Table 1), identified tensions in African Americans’ beliefs and attitudes about genomic research. The overarching theme of trust (or lack thereof) was present across studies, and influenced subsequent attitudes about genomic research and participation. However, even with concerns about trust, African Americans believed their participation in genomic studies was critical. These negative and positive beliefs informed their attitudes about participation in genomic studies. What follows is a summary of the literature highlighting tensions between distrust and the value of their participation.

**Table 1:** Studies included in Review.

References	Study design	Setting and population	Sample size	n(%) AA^∗^
Bates and Harris, 2004	Qualitative focus group study	Southeastern United States General population	*N* = 215	118(55)
Brewer et al., 2014	Quantitative cross-sectional survey study	Orlando, Florida at The Links, Incorporated 38th National Assembly Female Links Members	*N* = 381	381(100)
Buseh et al., 2013	Mixed methods; CBPR and focus group study	Wisconsin Genomic Initiative Community members	*N* = 21	21(100)
Bussey-Jones et al., 2010	Mixed methods telephone survey	North Carolina North Carolina Colorectal Cancer Study Database	*N* = 801	153(19%)
Cain et al., 2016	Quantitative cross-sectional survey study	Washington DC Metro Area Community members	*N* = 304	304(100)
Cohn et al., 2015	Qualitative exploratory study	Central Harlem, New York Community members	*N* = 46	39(89) 4(9) AA/ Hispanic 1(2) AA/ Native American
Dash et al., 2014	Mixed methods; focus group and cross-sectional survey study	Southeast/Southwest Washington, DC Community members	Focus groups (*n* = 41) Surveys (*n* = 321)	Focus groups 41(100) Surveys 234(73)
Diaz et al., 2008	Quantitative cross-sectional survey study	South Carolina State University Students	*N* = 200	200(100)
Drake et al., 2017	Qualitative focus group study	St. Louis, Missouri; Prostate Cancer Community Partnership Men with prostate cancer	*N* = 70	70(100)
Erwin et al., 2013	Mixed methods; focus group and cross-sectional survey study	Niagara Falls, New York Community members and Key informants	Key informant interviews (*n* = 9) Community focus groups (*n* = 21) Staff focus group (*n* = 5) Surveys (*n* = 64)	Community focus groups 13(62) Surveys 34(53)
Goldenberg et al., 2011	Mixed methods; computer assisted telephone interviewing system	Patients from Duke University, Johns Hopkins, University of Arizona, University of North Carolina, University of Utah	*N* = 1,193	192(16)
Hagiwara et al., 2014	Quantitative survey study	Detroit, Michigan Community members	*N* = 78	78(100)
Halbert et al., 2016	Quantitative survey study using vignettes	National sample of AA	*N* = 510	510(100)
Hoyo et al., 2003	Qualitative semi-structured interview and focus group study	North Carolina Community members	Focus groups (*n* = 46) Interviews (*n* = 9)	55(100)
Isler et al., 2013	Qualitative semi-structured interview study	North Carolina Community members	*N* = 91	72(79)
Jones et al., 2017	Quantitative cross-sectional survey study	Kansas City, Kansas Community members	*N* = 169	169(100)
Kraft et al., 2018	Qualitative focus group study using trigger videos	Northern California Patients at a large multispecialty practice	*N* = 122	23(18.9)
Lee et al., 2019	Qualitative focus group study using trigger videos	Northern California Patients at a large multispecialty practice	*N* = 122	23(18.9)
Luque et al., 2012	Qualitative focus group study	Tampa, Florida Community members	*N* = 95	33(34.7)
McDonald et al., 2014	Quantitative survey study	National sample of AA	*N* = 1,033	1,033(100)
McDonald et al., 2012	Qualitative focus group study	Philadelphia, Pennsylvania	*N* = 91	91(100)
Ochs-Balcom et al., 2011	Qualitative focus group study	Buffalo, New York Female breast cancer survivors	*N* = 14	14(100)
Skinner et al., 2015	Qualitative focus group study	Lenoir County, North Carolina Community members	*N* = 25	19(76)
Walker et al., 2014	Qualitative focus group study	Jackson, Mississippi Community members	*N* = 140	140(100)


*^∗^AA, African American.*

### Distrust

We found a shadow of historic and continued injustice cast across studies. Distrust was ubiquitous in all facets of the research enterprise and extended from members of the research and medical communities (Skinner et al., 2015; Drake et al., 2017; Kraft et al., 2018), to medical or research institutions (Drake et al., 2017; Kraft et al., 2018), and the conduct of research and science in general (Skinner et al., 2015). The Tuskegee Study of Untreated Syphilis frequently functioned as a historical referent for the distrust of biomedical research, particularly among African Americans (Hoyo et al., 2003; Bates and Harris, 2004; Cohn et al., 2015; Kraft et al., 2018). One study found African Americans were significantly more concerned that something like Tuskegee could happen again than white participants (Hagiwara et al., 2014). More specific to genetics, revelations about Henrietta Lacks, and more recent and local race-related abuses by researchers, raised concerns about trust, privacy and the benefits of genomic studies (Buseh et al., 2013; Drake et al., 2017; Kraft et al., 2018; Lee et al., 2019). The impact of race-related injustice was apparent in two multi-race studies that found distrust was more salient among African American participants compared with their white counterparts (Bussey-Jones et al., 2010; Hagiwara et al., 2014). The salience of race in historic injustices in the United States raised suspicions about researchers’ intentions, and the potential for race-based research to be used for maleficence ranging from racial discrimination to eugenics, or even genocide (Buseh et al., 2013; Isler et al., 2013; Kraft et al., 2018).

Distrust often was tied to fears about study processes and outcomes. Most frequently mentioned were fears of being experimented on or treated as a “guinea pig” or “lab rat” (Hoyo et al., 2003; Ochs-Balcom et al., 2011; Luque et al., 2012; Buseh et al., 2013; Erwin et al., 2013; Hagiwara et al., 2014; Walker et al., 2014), as was fear of exploitation (McDonald et al., 2012; Buseh et al., 2013). Several studies revealed beliefs that research is conducted at the expense of African Americans for the financial profit of those in power (Kraft et al., 2018; Lee et al., 2019), or to provide more effective treatments to white or privileged individuals (Luque et al., 2012; Halbert et al., 2016). Both African American and white participants in one study raised concerns about the possibility that genetic research could be used to discriminate against certain groups of people, with significantly more African Americans reporting that their concern about potential discrimination would influence their willingness to provide a blood sample for research (Goldenberg et al., 2011). Personal experiences with racial discrimination, and witnessing expanding health disparities in spite of medical advancements, added to beliefs that the medical and research communities were not trustworthy (Buseh et al., 2013). Among African Americans, increased distrust was significantly associated with reduced likelihood of biobank participation (McDonald et al., 2014; Halbert et al., 2016).

Despite concerns about trust and associated fears about participation, participants’ relationship with medical research was complicated (McDonald et al., 2012). Tensions existed between distrust of medical research and beliefs that African American participation in research is imperative (Bates and Harris, 2004; Ochs-Balcom et al., 2011; McDonald et al., 2012; Erwin et al., 2013; Hagiwara et al., 2014). In particular, participants described the necessity of African American participation in order to determine the efficacy and optimal dosing (i.e., PGX) and find more effective ways to treat and prevent diseases which frequently impact their race (Bates and Harris, 2004; Buseh et al., 2013; Erwin et al., 2013). In one study, neither concerns about exploitation nor distrust of medical research were associated with willingness to donate biological specimens for research (Hagiwara et al., 2014).

In contrast, some studies found African American participants trusted medical research and biobanks, and were favorable toward medical research (Hagiwara et al., 2014; Walker et al., 2014; Cain et al., 2016). More recent studies assessing African American community members’ knowledge, beliefs, and attitudes about medical and genomic research found study participants did not believe they would be taken advantage of or harmed by research focused on minorities (Cain et al., 2016; Jones et al., 2017). Female members of The Links Incorporated (a not-for-profit African American service organization) who believed research conducted in the United States was ethical were more willing to participate in genomic studies (Brewer et al., 2014).

The overarching theme of distrust was present in most, but not all studies. Even among those with high levels of distrust, the importance of African Americans’ participation in medical and genomic research was recognized. This dichotomy may explain why some studies found high levels of distrust and others did not. Participants’ divergent views may underlie an attempt to reconcile beliefs about distrust of medical research with the importance of their participation in medical research to avoid cognitive dissonance.

### Community Engagement

Participants described community engagement as one strategy to overcome distrust. Community members and leaders described how researchers often entered their community to obtain something from them, and then simply left (Buseh et al., 2013). Such interactions left the community feeling used, disrespected and engendered continued distrust (Buseh et al., 2013). Failing to engage community members prior to conducting studies was viewed as a barrier (Hoyo et al., 2003), whereas genuine engagement, care and communication were viewed as facilitators that created trust (Walker et al., 2014). “Authentic collaboration” is desired which means that researchers: (1) engage with community leaders and the community at the start of the project before major decisions are made, (2) ensure proper resources are available, (3) give credit to the communities, (4) maintain community engagement beyond the study, and (5) share study outcomes (Buseh et al., 2013; Cohn et al., 2015). Participants did not desire frequent contact, but they wanted to know how their participation contributed to the advancement of science (Cohn et al., 2015). Similarly, participants in a focus group study recommended working early on in the research process to improve relationships between institutions and community members citing existing strong relationships with local community hospitals as an example (Kraft et al., 2018).

### Awareness and Knowledge

Awareness and knowledge of genomics, or a desire to learn more were associated with favorable attitudes toward genomic studies and/or intentions to participate (Hoyo et al., 2003; Ochs-Balcom et al., 2011; Cohn et al., 2015; Jones et al., 2017). Conversely, lack of education, understanding, awareness or knowledge were associated with less favorable attitudes and lower intentions to participate (Hoyo et al., 2003; Bates and Harris, 2004; Ochs-Balcom et al., 2011; Skinner et al., 2015; Drake et al., 2017). Participants noted that information about research studies was not readily available in their communities, or that African Americans are often not approached or asked to participate (Drake et al., 2017).

Participants described opportunities to overcome low levels of awareness, such as providing educational sessions to ensure informed participation of African Americans (Buseh et al., 2013). Participants in another study suggested that researchers could learn as much from the community as the community could learn from researchers, and advocated for bidirectional educational efforts be bidirectional (Buseh et al., 2013). Similarly, research targeting the African American community was viewed as an opportunity for collaboration between researchers and community members (Cohn et al., 2015). Tying together trust and education, participants suggested that one way to prevent mistreatment of African Americans was for them to request additional information about research studies during recruitment (Bates and Harris, 2004; McDonald et al., 2012). Given this finding, researchers should anticipate that African Americans will have a greater need for information about study procedures than white participants do.

### Process of Study Conduct

Across studies, African Americans described their attitudes and beliefs about particular aspects of the research process including research team members and/or the associated institution, study procedures and safeguards, participation risk and compensation. We describe each category next.

### Face of the Study

African Americans reported in two studies that they were more likely to participate in research conducted by Historically Black Colleges (HBC) (Hoyo et al., 2003; Diaz et al., 2008). HBCs were viewed as more trustworthy, and participants believed the involvement of HBCs would ensure results and benefits from their participation would be returned to the African American community (Hoyo et al., 2003). Additionally, African Americans want to see African American physicians and/or researchers in leadership roles on the research team (Hoyo et al., 2003; Bates and Harris, 2004; Buseh et al., 2013; McDonald et al., 2014; Cain et al., 2016). It was believed researchers from shared racial backgrounds would be more likely to understand relevant cultural beliefs and experiences, and were viewed as more trustworthy (Hoyo et al., 2003; Bates and Harris, 2004; Buseh et al., 2013; McDonald et al., 2014; Cain et al., 2016; Kraft et al., 2018). In two studies African Americans reported that they were more likely to participate if the investigator was African American (Diaz et al., 2008; McDonald et al., 2014), and one study found a decreased likelihood of participation if the study was conducted by a predominately white college or a white investigator (Diaz et al., 2008).

Similarly, participants across several studies preferred information about genomic research or specific studies be delivered by African Americans (Diaz et al., 2008; Dash et al., 2014), particularly if the study was race specific (McDonald et al., 2014). Participants reported more favorable attitudes toward research, and an increased likelihood of enrollment when the study was introduced by a trusted other such as their physician, friends, family members, and/or community leaders (Hoyo et al., 2003; Diaz et al., 2008; Drake et al., 2017). Participants suggested that hearing about the research study within their community, and knowing others in their community who were involved in the study, would increase their likelihood of participation (Drake et al., 2017).

### Study Procedures and Safeguards

Given past injustices, African Americans held significant concerns about the use and accessibility of their data by other individuals or institutions. Due to racism and possible malevolent intent, across studies African Americans wanted to know specifically how their biological material might be used (Buseh et al., 2013; Hagiwara et al., 2014). Not knowing specifically how the specimen would be used was a barrier to participation (Dash et al., 2014). There were concerns about surreptitious use of genetic material for surveillance, to deny rights and privileges, in criminal investigations, and for other uses beyond the purpose of their original consent (Hoyo et al., 2003; Buseh et al., 2013; Cohn et al., 2015; Kraft et al., 2018). In addition to the aforementioned concerns, participants in one focus group study specifically mentioned concerns related to identity, cloning, and the use of their sample after death (Lee et al., 2019). In addition, not knowing who would have access to their personal information, and who might obtain access to their personal information (e.g., other medical entities like insurance companies) raised concerns, and in some cases, significantly decreased likelihood of participation (Ochs-Balcom et al., 2011; McDonald et al., 2014; Walker et al., 2014; Halbert et al., 2016). Across studies, transparency of study procedures and clear descriptions about safeguards to protect participant privacy were determined essential for participation. Specifically, African Americans want transparency and to know as much as possible about the purpose and rationale for the study, how their specimen would be used and by whom, and the safeguards in place to protect their privacy (Dash et al., 2014; Hagiwara et al., 2014; Skinner et al., 2015; Kraft et al., 2018). Furthermore, continued and ongoing communication about changes to study protocols, or changes to sample access, and the specific studies for which their sample would be used was important, as was maintaining the option to opt in or out of particular studies (Kraft et al., 2018; Lee et al., 2019).

### Participation Risk

One study identified that beliefs about the risk of participation were negatively associated with willingness to participate (Brewer et al., 2014), but another study found concerns about the risk of participation was only a consideration when making participation decisions (McDonald et al., 2012). Another study found African Americans were specifically worried about the possible contamination of equipment used for biospecimen collection (Hagiwara et al., 2014). Aside from risk, concerns about procedures primarily focused on invasiveness. Studies found participants least preferred studies where methods were viewed as invasive (Cain et al., 2016), and were more favorable toward participating in studies they believed were less invasive in terms of procedure, privacy, and resources (Hoyo et al., 2003; Diaz et al., 2008; Cain et al., 2016). Although one study found blood donation for participation in a genomic study to be minimally invasive (McDonald et al., 2012), other studies identified fear of needles or the donation of blood as a barrier to study participation (Ochs-Balcom et al., 2011; Dash et al., 2014; Drake et al., 2017).

Concerns about invasiveness included the expenditure of resources, specifically, cost and time. Participants in one study raised concerns about the potential personal costs of participating including costs associated with blood draws and genetic analysis (Skinner et al., 2015). Possible sustained participation in a longitudinal study evoked questions about the number of tasks and time required of participants (Hoyo et al., 2003; McDonald et al., 2012); participants were more favorable about participating in studies which only lasted a short period of time (McDonald et al., 2014). Participants viewed the distance they had to travel for study participation as a barrier to participation (McDonald et al., 2012; Cain et al., 2016). Any perceived expense to the participant such as cost or time for participation, including time that would be taken from work (Walker et al., 2014; Skinner et al., 2015) and transportation issues (McDonald et al., 2014; Halbert et al., 2016) were barriers to participation, unless compensation could be provided (Cain et al., 2016).

### Compensation

African Americans expected compensation for participants’ time for any study that required any type of time commitment, including travel. Compensation for such expenses were believed to increase participation (Erwin et al., 2013; Skinner et al., 2015; Cain et al., 2016; Drake et al., 2017; Jones et al., 2017), and in some cases, African Americans suggested profit sharing as a means for compensation (Buseh et al., 2013; Jones et al., 2017). However, across studies it was noted that the form of compensation did not always need to be direct participant payment. African Americans suggested that food, gas cards, healthcare and/or medication (Hoyo et al., 2003; Hagiwara et al., 2014; Drake et al., 2017), and even individual research results could be provided as a form of compensation (Skinner et al., 2015; Jones et al., 2017). Indeed, some studies found failure to provide research results to participants would prevent African Americans from participating (McDonald et al., 2014; Halbert et al., 2016).

### Individual Level Benefits and Drawbacks of Study Participation

African Americans’ interest in participating in genomic studies often was driven by beliefs about benefits for themselves, family members, or future generations. In some cases, individual benefit was broadly or unclearly defined (McDonald et al., 2012, 2014; Skinner et al., 2015; Jones et al., 2017). In other studies, individual benefit included the belief that participation in research meant they would receive better health care (Brewer et al., 2014). Participants across several studies believed they would derive individual benefit by learning more about their genetic risk, which, depending on the results, could act as a motivator for making positive lifestyle changes (Buseh et al., 2013; Skinner et al., 2015). Studies conducted with affected participants, or those already at risk for a specific disease, found increased interest in participation when the study could provide knowledge about the particular condition, for example, cancer (Ochs-Balcom et al., 2011; McDonald et al., 2014; Halbert et al., 2016), asthma (Jones et al., 2017), cardiovascular disease, or type 2 diabetes (Skinner et al., 2015).

Aside from personal benefit, African Americans across studies believed participation in genomic or biobank studies could provide insight into disease that would ultimately benefit their family members or future generations (Ochs-Balcom et al., 2011; McDonald et al., 2012; Dash et al., 2014; Walker et al., 2014; Skinner et al., 2015; Drake et al., 2017; Kraft et al., 2018). They also suggested benefits to family members or future generations could be indirect or much further into the future, such as helping researchers develop medicine that may be used by future generations (Dash et al., 2014).

Notably, two studies found participants did not believe there would be a personal benefit from participating in a research study, and did not believe they would be a benefactor of research outcomes (Halbert et al., 2016; Drake et al., 2017). African Americans believed they were unlikely to benefit personally from medical advancements due to insurance discrimination and the out of pocket costs associated with new pharmaceutical treatments (Halbert et al., 2016; Lee et al., 2019). In some cases, African Americans believe harm could come from finding out about a medical condition that they did not want to know about. As a result, in some studies, learning about personal genetic information was identified as a barrier to participation (Ochs-Balcom et al., 2011; Walker et al., 2014; Skinner et al., 2015; Drake et al., 2017; Jones et al., 2017).

### At the Community Level

The potential for genomic or biobank studies to improve health outcomes for their community was embraced by participants (Goldenberg et al., 2011; McDonald et al., 2012; Buseh et al., 2013; Walker et al., 2014; Cohn et al., 2015). Several studies highlighted participants’ beliefs that African American participation in medical research, and genomic research in particular, is essential as a means to address health issue of traditionally underserved populations as a means to reduce health disparities (Ochs-Balcom et al., 2011; McDonald et al., 2012; Isler et al., 2013; Skinner et al., 2015). African Americans in one study held the belief that their participation in today’s research would facilitate personalized medicine and more targeted prevention and treatment options for disease, for future generations of African Americans (Buseh et al., 2013). While African Americans were favorable toward race specific studies designed to improve health outcomes for their own race (Goldenberg et al., 2011; Ochs-Balcom et al., 2011; McDonald et al., 2014; Walker et al., 2014), results from one study found participants felt such studies were more likely to take advantage of or hurt minorities (Jones et al., 2017). Further, African Americans suggested that despite their participation and advances in medicine, they believed study results were unlikely to reach their community as a result of historic barriers to medical care (Luque et al., 2012). As a solution, African Americans suggested that any prevention or treatment innovations resulting from African American participation must be accessible and affordable for those community members (Buseh et al., 2013; Halbert et al., 2016). Yet, concerns were raised about whether genomic studies could address social determinants of health that are typically responsible for poor health outcomes, and are often ignored (Buseh et al., 2013).

Related to the belief that their participation could benefit their community, favorable views about participation in genomic studies or biobanks most frequently stemmed from altruistic beliefs. Participants believed participation in genomic studies would help future patients or people in general (McDonald et al., 2012; Skinner et al., 2015; Kraft et al., 2018). Caring for others and the benefit of participation to society were central to motivating participation (Brewer et al., 2014; Jones et al., 2017), despite concerns about trust (Bates and Harris, 2004).

## Discussion and Conclusion

Given favorable attitudes, but low participation rates, culturally appropriate and ethical messages about PGX studies that facilitate recruitment of African Americans are needed (Halbert et al., 2016). Trust has often been cited as the leading barrier to African American participation in health-related research (George et al., 2014; Luebbert and Perez, 2016; Hughes et al., 2017; Jones et al., 2017). Consistently, our review found that distrust in the healthcare system, medical research, organization, and researchers is a commonly held belief by many African Americans (Bates and Harris, 2004; Bussey-Jones et al., 2010; Hagiwara et al., 2014; McDonald et al., 2014; Cohn et al., 2015; Skinner et al., 2015; Halbert et al., 2016; Drake et al., 2017). We forward several suggestions to overcome distrust (see Table 2). First, meaningful and intentional community collaboration can demonstrate value and meaning for African American participants (Walker et al., 2014). Indeed, a systematic review conducted by Johnson et al. (2011) identified community-based strategies, such as engaging community leadership, as one method for improving recruitment of African Americans into genomic research. However, results from our review suggest researchers must move beyond simply contacting community leaders at the time of the study. Instead, researchers should engage in what participants called “authentic collaboration” from before the start of the research study and extending after the study as a means to foster trust, demonstrate respect and honor the value of community contributions (Buseh et al., 2013; Cohn et al., 2015). These findings are consistent with the success of other studies, which have used CBA as a method to improve recruitment of African Americans (Israel et al., 1998; Vadaparampil and Pal, 2010; Kiviniemi et al., 2013; Ochs-Balcom et al., 2015; McNeill et al., 2018).

**Table 2:** Summary of Beliefs and Attitudes and Message Design Opportunities.

Beliefs and attitudes	Message design opportunities
**Barriers to recruitment**	
**Distrust** – of researchers, universities or health care organizations, science and medicine at large	1. Establish relationship with community members prior to beginning research study and engage them in recruitment design efforts 2. Consider engaging African American community members, including other research participants and community health care workers, as the senders/disseminators of recruitment messages 3. Engage African American study team members as senders/disseminators of recruitment messages 4. Provide a clear description of study purpose, procedures, who will be able to access their data and privacy safeguards in place 5. Messages about the use of participant data should be clearly detailed 6. Describe how information from the study may impact health care for the African American population 7. Any and all forms of compensation should be clearly described in any study asking for participants’ time, including travel time
**Lack of Education** – about research studies and genetics created less favorable attitudes about participation	1. Outreach efforts should focus on providing more information about genomic studies more broadly 2. Delivering in-person education may be advantageous because researchers can address additional questions or concerns on the spot, and at the same time engage with and learn from the population 3. Combine educational messages with messages that describe use of data and standard privacy protections that are in place 4. Messages should provide detailed information about research purpose, processes and outcomes
**Facilitators of recruitment**	
**Participation** – beliefs that African American participation is necessary and essential	1. Messages should emphasize the importance of African American participation for their community 2. When appropriate, messages should describe any potential individual level benefit from participation in the study 3. When appropriate, messages should describe any potential future benefit to family members 4. When appropriate, messages should describe any potential future benefit for the African American community 5. Messages about altruism should be included in recruitment efforts


Our review also identified lack of knowledge or awareness about genomic studies as an overarching barrier (Hoyo et al., 2003; James et al., 2008; Skinner et al., 2008; Ochs-Balcom et al., 2011; Drake et al., 2017). However, educational interventions have demonstrated little impact on attitudes or beliefs, thus suggesting messages that address existing attitudes and beliefs in addition to providing education may be more effective at addressing African Americans’ concerns about participation in genomic studies (Skinner et al., 2008; Halverson and Ross, 2012). Furthermore, it could be argued that beliefs about the trustworthiness of research scientists or institutions (Luque et al., 2012; Erwin et al., 2013; Hagiwara et al., 2014; Walker et al., 2014) impact African Americans’ expectations for research participation. For example, African Americans concerns about being experimented on or exploited explain why they want complete transparency about study protocols and data sharing practices (Dash et al., 2014; Hagiwara et al., 2014; Skinner et al., 2015). As such, messages that are transparent and clearly describe the study protocol may reduce mistrust as a barrier. Based on our review, messages for African Americans about genomic studies should provide substantial information about the study purpose and procedure and describe processes and measures in place to safeguard their privacy. Previous research found that messages which intentionally highlight procedures and security are more likely to overcome concerns related to privacy and outcomes (McQuillan et al., 2006; George et al., 2014; Luebbert and Perez, 2016; Hughes et al., 2017; Jones et al., 2017).

Contrary to the belief that minority populations are not interested in participating in research studies, our review found African Americans were highly interested in participating (Wendler et al., 2005; Horowitz et al., 2017; Jones et al., 2017). Studies in our review indicated African Americans believed their participation in medical research was crucial for the advancement of science (Bates and Harris, 2004; McDonald et al., 2012; Erwin et al., 2013; Hagiwara et al., 2014). Thus, researchers should devote more attention to facilitators of African American participation in medical research. Specifically, as identified in our review, messages that highlight altruism or benefit for one’s community and recognize the importance of including minority populations may promote participation in clinical studies of African Americans (George et al., 2014; Hughes et al., 2017; Jones et al., 2017).

Ultimately, one goal of PM research is to reduce health disparities (Collins and Varmus, 2015; Khoury et al., 2016). In particular, PGX uses personal genomic data to inform optimal tailoring of pharmaceutical agents to prevent adverse drug interactions (Perera et al., 2014). Despite the individualized focus of PGX, efforts require a population-based approach to better understand inter-population and intrapopulation diversity (Bonham et al., 2016; Khoury et al., 2016). This review drew upon existing literature to provide a consolidated overview of African American’s beliefs and attitudes toward genomic research. This information can inform recruitment strategies and messages that may increase African American participation in genomic studies, and PGX studies in particular. Future research testing the message strategies identified in this review are needed to continue to understand best practices for communicating genomic research with the African American population. Additionally, future studies should explore African Americans’ beliefs and attitudes regarding PGX studies. Such knowledge may contribute to the advancement of PM among minority populations.

## Author Contributions

CS, SR, and MP conceptualized the study. CS, SR, and CM-F devised the methods, conducted the literature search, reviewed the literature, and conducted the analysis. CS and SR drafted the manuscript. MP reviewed and edited the manuscript.

## Conflict of Interest Statement

The authors declare that the research was conducted in the absence of any commercial or financial relationships that could be construed as a potential conflict of interest.
